# Cat-scratch disease manifesting as uveitis and binocular fundus nodular lesions: a case report

**DOI:** 10.1186/s12886-023-03063-4

**Published:** 2023-08-07

**Authors:** Hao Hong, Tianxi Li, Ye Ying, Qi An, Hu Liu, Kang Liang

**Affiliations:** 1 Department of Ophthalmology, Nanjing hospital, Nanjing University of Traditional Chinese Medicine, Nanjing, China; 2 Department of Ophthalmology, The First Affiliated Hospital with Nanjing Medical University, 300 Guangzhou Road, Nanjing, 210029 China

**Keywords:** Cat-scratch disease, Bartonella, Metagenomics next-generation sequencing (mNGS), Ocular manifestations, Fundus

## Abstract

**Background:**

Cat-scratch disease typically presents with various ocular manifestations such as uveitis, vitritis, retinitis, retinochoroiditis, and optic neuritis. However, fundus nodular lesions was rarely reported. In our study, we reported a case of Cat-Scratch disease with binocular fundus nodular lesions.

**Case presentation:**

An 11-year old male presented with uveitis in the right eye and bilateral fundus nodular lesions after indirect contact with unvaccinated cats. Comprehensive ancillary examinations including wide-angle fundus photography, ultrasonography, fluorescein fundus angiography, optical coherence tomography, and orbital magnetic resonance imaging were performed to elucidate the multidimensional features of the binocular lesions. Metagenomics next-generation sequencing was utilized to confirm the diagnosis of Cat-scratch disease. The patient’s condition showed improvement after a 6-month combination treatment regimen involving systemic administration of doxycycline hyclate and methylprednisolone tablets, as well as local application of mydriatic and corticosteroid eye drops.

**Conclusions:**

We firstly reported a case of Cat-scratch disease presenting simultaneously with uveitis and fundus nodular lesions caused by Bartonella henselae infection in a child. Timely diagnosis and treatment with antibiotics and corticosteroids showed promising outcomes for the prognosis of these ocular disorders.

## Background

Cat-scratch disease (CSD) is a self-limiting infectious disease primarily caused by Bartonella henselae (B. henselae) infection. Ocular manifestations of CSD encompass a spectrum of conditions, including uveitis, neuroretinitis, vasculitis, retinal vessel occlusions, retinal detachment, and Parinaud’s oculoglandular syndrome (POGS). Previous reports have predominantly described CSD cases presenting as unilateral inflammatory ocular disorders. In this article, we presented an unusual pediatric case of CSD with simultaneous bilateral involvement, characterized by a subretinal nodular lesion and panuveitis in the right eye, as well as an isolated fingerlike projection extending into the vitreous cavity in the left eye.

## Case presentation

An 11-year-old male patient consulted in the Department of Ophthalmology at the First Affiliated Hospital with Nanjing Medical University on December 25, 2021, with a chief complaint of blurry vision persisting for 5 days. The patient reported a preceding history of sneezing, a runny nose, and fever, with a peak temperature of 38.9℃, which resolved following the administration of antipyretic medication approximately 10 days prior to the onset of visual disturbances. Subsequently, the patient experienced progressive deterioration of visual acuity and the presence of blind spots in the right eye, accompanied by mild photophobia, tearing, and conjunctival hyperemia. No ocular discomfort was complained in the left eye. The patient resided in a rural area and had regular contact with 2 cats and 2 dogs, none of which had been vaccinated before.

### Physical and ocular examination

**Fig. 1:**
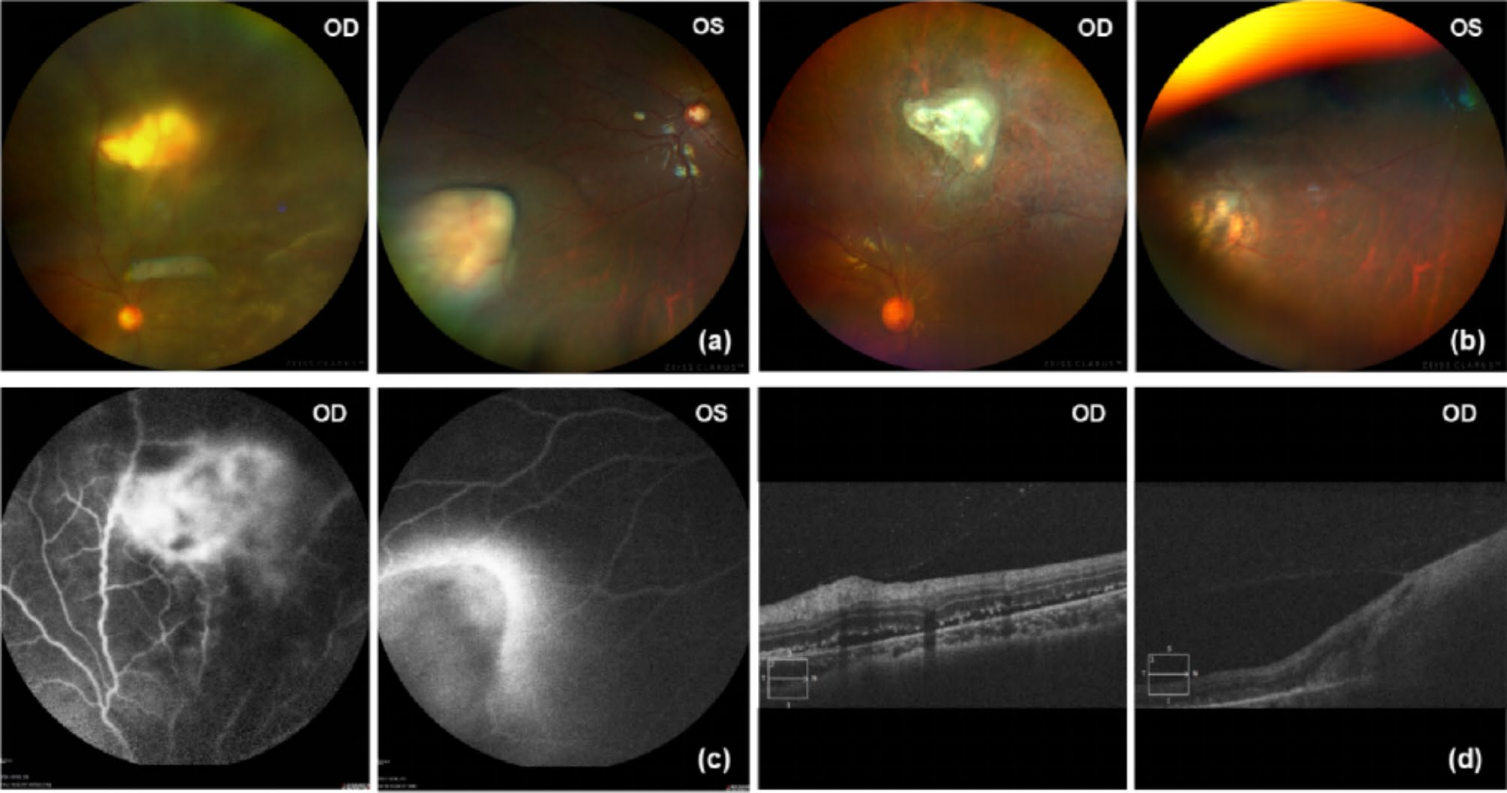
Fundus examinations. **(a)**: Wide-angle fundus photography prior to treatment. **OD**: subretinal nodular lesion in the right eye; **OS**: isolated fingerlike projected lesion extending into the vitreous cavity in the left eye. **(b)**: Wide-angle fundus photography 6 months after treatment. OD: lesion of the right eye appeared dissolution and scarring; **OS**: projected lesion of the left eye was resolved with pigmentation. **(c)**: Fluorescein fundus angiography prior to treatment. **(d)**: Optical coherence tomography prior to treatment

There was no enlargement of superficial lymph nodes throughout the body. Ocular examination revealed that the uncorrected visual acuity was 20/63 and 20/20, the best corrected visual acuity (BCVA) was 20/63 and 20/16 in his right eye and left eye respectively. Intraocular pressure (IOP) was 14.7 mmHg in the right eye and 13.0 mmHg in the left eye. The right eye showed a clear cornea, 2 + aqueous cells and flare, pigmented cells on the lens surface, and massive vitreous opacity. Fundus examination revealed a yellowish white subretinal nodular lesion about 6 prism diopters above optic disc, with vascular tortuosity and vessel dilatations around. Furthermore, exudative retinal detachment was evident within and around the lesion, extending to the nasal and inferior regions of the retina. The anterior segment and vitreum appeared normal in the left eye. Fundus examination of the left eye disclosed an isolated pale fingerlike projected lesion extending into the vitreous cavity. This lesion was located close to the inferior nasal ora serrata region. The surrounding retina in the left eye exhibited a normal appearance (Fig. 1a).

### Ancillary examination

**Fig. 2:**
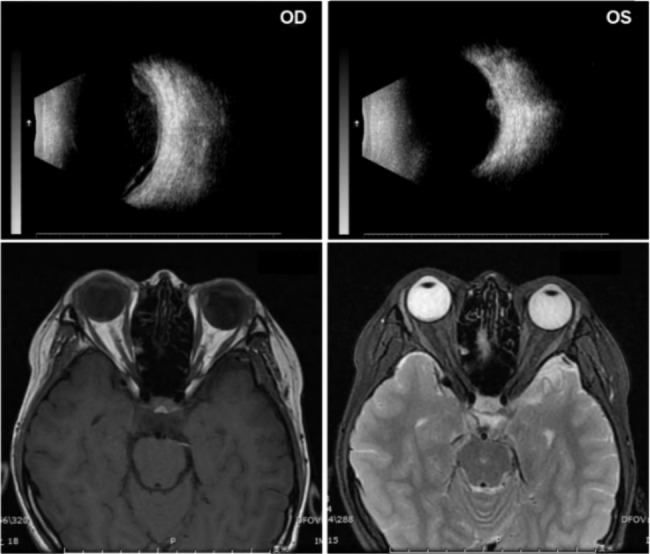
Ancillary Examination prior to treatment. **(a)**: Ultrasonography; **(b)**: Orbital Magnetic Resonance Imaging

In the ultrasonography B-scan of the right eye, a hyperechoic membrane was observed floating within the vitreous cavity. The left eye displayed a small number of punctate low-to-medium echoes in the vitreous cavity and a medium fingerlike echo that was attached to the posterior eyeball (Fig. 2a). In the fluorescein angiography (FFA) examination, in the right eye, early-stage lesion exhibited mottled hyperfluorescence, mid-stage showed fluorescence leakage, and late-stage demonstrated diffuse leakage with pooling of fluorescence in the subretinal space. The peripheral retinal vessels surrounding the lesions appeared tortuous and dilated. In the left eye, fingerlike lesion covered fluorescence in the early stage and manifested hypofluorescence with leakage around at later stage (Fig. 1c).

Optical coherence tomography (OCT) indicated the separation of the retinal neuroepithelium and pigment epithelium in and around the lesion in the right eye. Peripheral retina, where projected lesion in the left eye located was beyond the scanning range of OCT. (Fig. 1d). According to Orbital Magnetic Resonance Imaging (MRI) (Fig. 2b), a projection with clear margins, approximately 6.3 mm * 2.6 mm in size, was seen in the medial wall of left orbit, with slightly high signal on T1-weighted imaging (T1WI) and mixed signal on T2-weighted imaging (T2WI). Orbital computed Tomography scan (CT) and skull CT indicated no neoplasm or space occupying lesion. Chest CT revealed a 3 mm solitary pulmonary nodule in the right upper lung field, meanwhile abdomen CT indicated multiple small mesenteric lymph nodes.

### Laboratory examination

Routine blood tests showed a decrease in the neutrophil ratio (28.4%↓) and an increase in leukocytes (62.4%↑). Other parameters such as white cell count, eosinophils, hypersensitive C-reactive protein (hs-CRP), serological tests for syphilis, human immunodeficiency virus (HIV), tuberculosis, detection of worm eggs in stool smears, liver enzymes, kidney function, and angiotensin-converting enzyme were within normal range. Intraocular fluid examination revealed a significant increase in the level of IL-6, IL-8 and VCAM in the aqueous humor of the right eye. Polymerase Chain Reaction (PCR) tests for Herpes simplex virus (HSV), varicella-zoster virus (VZV), and toxoplasmosis (TOX) yielded negative results, indicating no relevant infection. Enzyme linked immunosorbent assay (ELISA) for specific antibodies indicated a slight increase in HSVIgG and VZVIgG levels, while Toxoplasma IgG and Toxocara IgG levels remained within normal limits (Table 1). The metagenomics next-generation sequencing (mNGS) conducted on vitreous fluid samples from the right eye identified B. henselae with a relative abundance of 0.71%.

**Table 1:** The detection of pathogenic microorganisms and inflammatory factors in aqueous humor

Parameter	Result	Reference range		Parameter	Result	Reference range
IL-6 (pg/ml)	24780.7	1.0 ~ 50.0		TOX-DNA in intraocular fluid (copy/ml)	0	
IL-8 (pg/ml)	329.3	0 ~ 20.0		TOX-IgG in intraocular fluid (IU/ml)	1.42	< 4
VCAM (pg/ml)	32257.0	200 ~ 1000		Toxocara-IgG in intraocular fluid (U)	1.31	< 3
HSV (copy/ml)	0	< 1E + 03		TOX-IgG in serum (IU/ml)	1.92	< 20
HSV-1 (copy/ml)	0	< 500		Toxocara-IgG in serum (U)	7.15	< 9
HSV-2 (copy/ml)	0	< 500		Total IgG in intraocular fluid (ng/ml)	993.0	
VZV (copy/ml)	0	< 5E + 02		Total IgG in serum (ng/ml)	21225.0	
HSV-IgG (U/ml)	114.81	< 9		Goldmann-Witmercoefficient of toxoplasmosis	15.81	0 ~ 2
VZV-IgG (U/ml)	5.23	< 16		Goldmann-Witmercoefficient of toxocara	3.92	0 ~ 2
				Total IgE in intraocular fluid (ng/ml)	0	
				Total IgE in serum(ng/ml)	16.4	

Based on these findings, we diagnosed the case as: (1) Cat scratch disease (both eyes); (2) panuveitis (right eye). The patient’s treatment regimen commenced with oral administration of doxycycline hyclate at a dosage of 100 mg twice daily for a duration of 28 days. In addition, a tapered dose of methylprednisolone tablets was prescribed, starting at 16 mg once daily for 7 days, followed by a reduction to 8 mg once daily for the subsequent 7 days, and finally to 4 mg once daily for the remaining 7 days. Furthermore, the patient received local application of mydriatic and corticosteroid eye drops specifically targeting the right eye.

At the 7-day follow-up after initiating treatment, the patient reported subjective improvement in visual acuity and a reduction in blind object floaters in the right eye. Notably, there was a significant decrease in pigmented cells on the front surface of the crystal. Additionally, the vitreous opacity showed signs of alleviation, the subretinal nodular lesion appeared flattened, and the exudative retinal detachment demonstrated improvement in the right eye. In the left eye, the previously observed isolated fingerlike projected lesion resolved, leaving behind a small amount of grey-white punctiform exudate. After one month of treatment, BCVA reached 20/16 in both eyes, and IOP measured 13.0mmHg and 14.3mmHg in the right and left eyes, respectively. The right eye displayed a tranquil anterior segment, clear vitreum, and normal retina, with dissolution of the nodular lesion, improvement in vascular tortuosity, and resolution of subretinal fluid. In the left eye, the size of the projection remained similar to that observed one week after treatment initiation. Six months after treatment, BCVA was 20/16 in both eye. IOP was 15.7mmHg and 16.3mmHg in the right and left eyes, respectively. Both eyes exhibited blue-grey atrophy without vascular tortuosity at the site of the lesion (see Fig. 1b).

## Discussion and conclusions

This study presents a unique case of an 11-year-old male who presented with uveitis in the right eye and binocular nodular lesions on the fundus following indirect contact with unvaccinated cats. Notably, this is the first documented case of CSD manifesting as subretinal nodules. To aid in disease diagnosis, we employed next-generation sequencing, an emerging technology in the field. Additionally, we conducted a comprehensive documentation of the clinical course and outcome of the disease.

Bartonella, which are aerobic gram-negative rods, have been identified as the causative agents of various zoonotic infectious diseases. Cats serve as the primary reservoir for B. henselae, with transmission occurring through blood-sucking arthropod animals, leading to both cat-to-cat and cat-to-human transmission [1]. Song [2] tested anti-B. henselae IgG in serum from Chinese individuals, revealing a positive rate of 9.68% among the 1260 samples analyzed. The estimated incidence of CSD is approximately 6.4 per 100,000 individuals per year in southern United States and 0.93 per 1000,000 individuals per year in Spain [3, 4].

CSD typically manifests as red papules at the site of injury and proximal regional lymphadenitis. Approximately half of the cases present with systemic manifestations, such as fever and night sweats. Immunocompromised individuals may experience severe complications, including thrombocytopenic purpura, encephalitis, osteomyelitis, and endocarditis [5]. Ocular manifestations are observed in around 5–10% of CSD patients and tend to appear 2–3 weeks after the onset of systemic symptoms. It is important to note that some CSD patients with ocular complications do not have a history of cat scratch or insect bite. An alternative explanation for contracting CSD is the contamination of the conjunctiva by infected flea feces [6]. Although the reported case did not disclose a history of cat scratch or bite, the patient had a habit of rubbing his eyes after coming into contact with cats. Therefore, we hypothesized that transmission in this case might have occurred through inoculation of contaminated flea feces from hands to eyes.

Kisaa [7] reviewed literature concerning ocular complications of CSD between January 1950 and September 2018. Ocular manifestations of CSD are diverse and usually monocular, among which neuroretinitis and POGS are most common. In the patient described here, subretinal nodular lesion, panuveitis, and regional exudative retinal detachment were detected in the right eye, and isolated fingerlike projected lesion was observed in the left eye. To our knowledge, this is the first case manifesting binocular fundus nocular lesions in CSD patients. According to previous report, granulomatous lymphadenitis could be frequently seen in biopsy of enlarged lymph nodes in CSD [8, 9]. In this case, either intraocular or extraocular biopsies would cause unacceptable damage to the patient. Therefore, we lacked pathological evidence to confirm whether the nodular lesion in this patient is granulomatous. Concomitant uveitis in the right eye was probably an evidence for central necrosis of lesion or immune inflammatory response.

Considering the diverse range of symptoms associated with CSD, laboratory examinations play a crucial role in reaching a definitive diagnosis. Traditional diagnostic methods, such as serological tests, exhibit limited specificity and sensitivity due to the challenging growth conditions of the host strain and the difficulty in isolating B. henselae from serum [10]. However, the emergence of mNGS has revolutionized the field by enabling comprehensive analysis of microbial and host genetic material (DNA and RNA) in patient samples, providing valuable sequence information through a single run with minimal sample input [11]. Although the application of mNGS in Bartonella infection is still relatively rare, this innovative approach is gaining momentum in the diagnosis of infectious diseases [12, 13]. In the presented case, we adopted a pioneering approach by performing mNGS on vitreous fluid samples, which revealed the presence of the B. henselae with a relative abundance of 0.71%, warranting significant attention. Emerging mNGS will significantly enhance the diagnostic workflow for infectious diseases, benefiting a larger number of patients. However, the prohibitively high cost of mNGS renders it a secondary option following the initial visit. We suggest that until the cost of mNGS is reduced, physicians should not prioritize mNGS as the initial diagnostic choice for most patients with CSD who present with a clear medical history and typical signs. Nonetheless, for a minority of patients with clinically atypical intraocular diseases, when conventional diagnostic methods fail to yield a definitive diagnosis, we recommend that physicians proceed with vitreous tapping and subsequent mNGS testing.

Despite being described as self-limited in immunocompetent individuals, cat scratch disease (CSD) still carries the potential for visual function impairments [14]. The management of CSD-associated ocular infections lacks a strong evidence base, primarily relying on case reports or case series due to the absence of clinical trials. In the presented case, the patient exhibited a favorable response to pharmacological therapy consisting of doxycycline hyclate and corticosteroids, resulting in significant improvements in both visual acuity and ocular structural integrity. We attributed the favorable visual prognosis in this case to: (a) the patient being in the early stage of the disease, (b) the lesion not severely affecting the macula and optic nerve, and (c) the patient receiving effective and standardized treatment after a definitive diagnosis.

There are alternative approaches in the diagnosis and treatment of the patient. Vitrectomy is a viable diagnostic and therapeutic option. Once the patient experiences worsening vitreous opacity, rapid progression of retinal lesions, or retinal detachment, we would promptly proceed with vitrectomy as a treatment. However, in the diagnostic process of this case, ocular lesions progressed slowly, allowing us to implement a treatment plan that is safer and less damaging to the patient. Considering the potential risk of sub-tenon triamcinolone acetonide injection exacerbating infectious diseases before establishing definitive diagnosis, it is not among our alternative treatment options. Even if the patient is diagnosed with non-infectious uveitis, there’s no evidence about the better choice of blindly administering sub-tenon triamcinolone acetonide injection compared to the standardized corticosteroid pulse therapy and immunosuppressive treatment. Therefore, we do not recommend performing sub-tenon triamcinolone acetonide injection in such patients before a definitive diagnosis is established.

In conclusion, we reported a rare case of CSD characterized by binocular fundus nodular lesions. The patient’s prognosis demonstrated the favorable therapeutic effects of antibiotics and corticosteroids in the management of CSD.

## Data Availability

The data and materials are available in the manuscript.

## Abbreviations

CSD: Cat-scratch disease
B. Henselae: Bartonella Henselae
BCVA: Best corrected visual acuity
IOP: Intraocular pressure
FFA: Fluorescein fundus angiography
OCT: Optical coherence tomography
MRI: Magnetic resonance imaging
T1WI: T1-weighted imaging
T2WI: T2-weighted imaging
CT: Computed tomography scan
hs-CRP: Hypersensitive C-reactive protein
HIV: Human immunodeficiency virus
PCR: Polymerase chain reaction
HSV: Herpes simplex virus
VZV: Varicella-zoster virus
TOX: Toxoplasmosis
ELISA: Enzyme linked immunosorbent assay
mNGS: Metagenomics next-generation sequencing
POGS: Parinaud’s oculoglandular syndrome
